# Clarithromycin in Early Pregnancy and the Risk of Miscarriage and Malformation: A Register Based Nationwide Cohort Study

**DOI:** 10.1371/journal.pone.0053327

**Published:** 2013-01-02

**Authors:** Jon Trærup Andersen, Morten Petersen, Espen Jimenez-Solem, Kasper Broedbaek, Nadia Lyhne Andersen, Christian Torp-Pedersen, Niels Keiding, Henrik Enghusen Poulsen

**Affiliations:** 1 Laboratory of Clinical Pharmacology, Copenhagen University Hospital Rigshospitalet, Copenhagen, Denmark; 2 Department of Clinical Pharmacology, Bispebjerg Hospital, Copenhagen, Denmark,; 3 Mental Health Centre Copenhagen, Copenhagen, Denmark; 4 Department of Cardiology, Gentofte University Hospital, Hellerup, Denmark; 5 Faculty of Health Sciences, University of Copenhagen, Copenhagen, Denmark; The University of Edinburgh, United Kingdom

## Abstract

**Background:**

The antibiotic clarithromycin has been associated with fetal loss in animals and a study has found a doubling in the frequency of miscarriages among women using clarithromycin in pregnancy. The aim of the study was to investigate whether clarithromycin use in early pregnancy was associated with an increased risk for miscarriages and major malformations.

**Methods:**

We conducted a nationwide cohort study including all women in Denmark with a known conception between 1997 and 2007. The Fertility Database was used to identify all women giving birth and the National Hospital Register was used to identify all women with a record of miscarriage or induced abortion. Prescription data was obtained from the National Prescription Register. The primary outcome was the number of miscarriages and offspring with major congenital malformations among users of clarithromycin compared to non-users.

**Results:**

We identified 931 504 pregnancies (705 837 live births, 77 553 miscarriages, and 148 114 induced abortions). 401 women redeemed a prescription of clarithromycin in the first trimester of which 40 (10.0%) experienced a miscarriage and among the live born nine (3.6%) had offspring with malformations. The hazard ratio (HR) of having a miscarriage after exposure to clarithromycin was 1.56 (CI95% 1.14–2.13). There was no increased hazard of having a miscarriage when being exposed to penicillin or erythromycin. There was no increased prevalence (OR = 1.03 (CI95% 0.52–2.00)) of having offspring with malformations after exposure to clarithromycin.

**Conclusions:**

We found an increased hazard of miscarriage but no increased prevalance of having offspring with malformations among women redeeming a prescription of clarithromycin in early pregnancy. This is supported by previous studies in animals and humans. However, further research is required to explore the possible effect of treatment indication on the associations found.

## Introduction

Clarithromycin is a macrolide antibiotic used to treat common infections including respiratory tract infections, skin infections and helicobacter pylori infections. Only limited data are available concerning the effect of clarithromycin on the human fetus when used in pregnancy.

Animal studies have shown that clarithromycin can induce fetal loss in rabbits and monkeys when used in very low dosages and in high dosages, respectively. [1] One observational study concerning pregnant women showed a doubling of the number of miscarriages in women exposed to clarithromycin in early pregnancy compared to a match control group. [2] There is limited knowledge concerning the risk of congenital malformations among women exposed to clarithromycin during pregnancy.

Based on the current knowledge clarithromycin is not recommended for use in pregnancy. However, since use of clarithromycin is used in very common conditions and only half of pregnancies are planned a substantial number of women risk exposure to clarithromycin in early pregnancy. [3].

We therefore conducted a nationwide cohort study testing the hypothesis that use of clarithromycin in the first trimester is associated with miscarriage. Furthermore, we investigated whether there is an association between use of clarithromycin in the first trimester and major congenital malformations.

## Methods

We identified all registered pregnancies in Denmark with a conception between 1 January 1997 and 31 March 2007 (n = 934 480). We excluded 2976 pregnancies due to coding errors.

From the Danish Fertility Database all live births with a conception date in the study period were identified. Using the National Hospital Register we identified all registered cases of miscarriages and provoked abortions (O02, O03, O04, O05 or O06 according to the International Classification of Diseases 10^th^ Danish revision). Information on major malformations was obtained from the National Hospital Register. All major malformations and subgrouping are according to the European Surveillance of Congenital Anomalies (EUROCAT) classification system guide 1.3. [4]

The Danish Fertility Database consists of individual-level data on the mother and father, including a unique identification number, age, previous births and abortions, as well as birth weight and length, death and cause of death, sex and gestational age of the offspring. The time of conception is based on ultrasound estimates or information of the date of last menstruation. More than 99.5% of births in Denmark since 1978 are registered in the Danish Fertility Database. [5], [6] The National Hospital Register contains information on all hospitalizations in the country, including admittance data and discharge diagnosis. [7], [8] It holds more than 99% of discharge records from all Danish hospitals. [9] Since 1997, information on gestational length has been added to diagnoses of provoked abortion and miscarriage.

Information on prescription medication use was collected from the National Prescription Register (the Register of Medicinal Product Statistics). [10], [11] Exposure was defined as redemption of a prescription of a drug containing clarithromycin (Anatomical Therapeutic Chemical Classification (ATC) J01FA09) for systemic use.

The register contains individual-level data on all prescribed drugs dispensed at all pharmacies in Denmark since 1995. Pharmacies are required by law to register prescriptions and this activity is coupled with reimbursement of expenses from the state, which ensures highly accurate prescription data. Completeness has previously been estimated to be 97.5%. [12] The register has no information on indication of treatment or over-the-counter drugs.

The infection treated with clarithromycin, could itself induce miscarriages. To test for this possible confounding by indication we compared the hazard of miscarriage for redeeming a prescription of clarithromycin with the hazard of miscarriage when redeeming a prescription of phenoxymethylpenicillin (ATC J01CE02) for systemic use or erythromycin (ATC J01FA01) for systemic use in the first trimester. Clarithromycin is furthermore used in treatment of helicobacter pylori infections in combination with proton-pump inhibitors (PPI) (ATC A02BC) and amoxicillin (ATC J01CA04). We therefore tested whether redemption of prescriptions on these drugs in the first trimester was associated with miscarriages. Furthermore we compared the risk of miscarriage of women exposed to clarithromycin directly against the risk of women exposed to the four above mentioned drugs.

To exclude the importance of other characteristics of women using clarithromycin during pregnancy, we compared the result with the hazard of dispensing clarithromycin in the 12 weeks before pregnancy.

### Statistics

All data management and analyses were performed using SAS software, version 9.2 (SAS Institute Inc., Cary, NC, USA).

Cox proportional hazard regression models with first trimester exposure to clarithromycin as a time dependent variable and time from conception to miscarriage as outcome. Time to birth or induced abortion was considered as censoring variables. Prescriptions redeemed after miscarriage or censoring was not included in the analyses.

A priori, an unadjusted model was planned in addition to a model adjusted for maternal age (five categories, <20, 20–24, 25–29, 30–34 and ≥35 years), the number of previous miscarriages (four categories, 0, 1, 2, ≥3 miscarriages), income (categorized as quartiles) and education (four categories).

Two logistic regression models were used to estimate the ratio of malformations among women redeeming a prescription of clarithromycin in the first trimester compared to unexposed. The first model was unadjusted and the second adjusted for maternal age (five categories, <20, 20–24, 25–29, 30–34 and ≥35 years), the number of previous births (four categories, 0, 1, 2, ≥3), income (categorized as quartiles) and education (four categories).

Data on maternal age, previous registered miscarriages, previous births and income had less than 1% missing values. If information on highest achieved educational level was missing, data from the following year was used. Data on educational level was missing for 3.4% of the records.

For all analyses, a two-sided value of p<0.05 was considered statistically significant, and all hazard ratios and odds ratios are presented with 95% confidence intervals.

### Ethics

All data were linked in computers held by Statistics Denmark and were made available with encrypted personal information. This ensured that no individuals could be identified. In Denmark The Act on Processing of Personal Data does not require ethical permission or obtained written informed consent for anonymised retrospective register studies. The Danish Data Protection Agency approved the study (No. 2008-41-2517).

We report our findings according to strengthening the reporting of observational studies in epidemiology (STROBE). [13].

## Results

We included 931 504 pregnancies identified within the study period. 77 553 (8.3%) miscarriages, 148 114 (15.9%) induced abortions and 705 837 (75.8%) live births.

Women redeeming a prescription of clarithromycin in the study period were older (p = 0.003) and had a lower educational level (<0.0001) compared to unexposed women.

### Miscarriage

In the 401 pregnancies exposed to clarithromycin in the first trimester, 40 (10.0%) of the pregnancy outcomes were recorded as miscarriages, compared to 77 513 (8.3%) in the unexposed group. We found a hazard ratio of 1.66 (CI95% 1.22–2.26) of having a miscarriage when being exposed to clarithromycin. Adjusting for age, number of previous miscarriages, educational level and income the hazard was 1.56 (CI95% 1.14–2.13).

### Major Congenital Malformations

Among the 401 women exposed to clarithromycin in the first trimester 253 resulted in live births. Among these, nine children were diagnosed with a major malformation (3.6%) compared to 24 808 (3.5%) among children born by unexposed mothers (table 1). We found an unadjusted odds ratio of 1.01 (CI95% 0.52–1.97) of having a child diagnosed with a major malformation when exposed to clarithromycin in the first trimester compared to unexposed women, and an adjusted odds ratio of 1.03 (CI95% 0.53–2.00). There was no significant difference in the number of major malformation according to the EUROCAT grouping system between women exposed to clarithromycin and unexposed women (table 2).

**Table 1:** Basic characteristics.

	All pregnant women	
	Clarithromycin use n = 401	No clarithromycin use n = 931 103
Age (years)		
<20	15 (3.7%)	30 811 (3.1%)
20–24	50 (12.5%)	120 727 (13.0%)
25–29	97 (24.2%)	296 369 (31.8%)
30–34	139 (34.7%)	308 991 (33.2%)
≥35	100 (25.0%)	174 205 (18.7%)
Education		
Low	199 (50.5%)	343 774 (37.8%)
Medium	103 (26.1%)	270 084 (29.7%)
High	69 (17.5%)	264 207 (29.1%)
No information	23 (5.8%)	31 157 (3.4%)
Income		
Lowest quartile	105 (26.2%)	232 706 (25.0%)
Low quartile	116 (28.9%)	232 639(25.0%)
Medium quartile	96 (23.9%)	232 765 (25.0%)
High quartile	84 (21.0%)	232 993 (25.0%)
Number of previous miscarriages		
0	321 (80.2%)	770 664 (82.3%)
1	62 (15.5%)	129 360 (13.9%)
2	13 (3.2%)	24 315 (2.6%)
≥3	5 (1.3%)	6764 (0.7%)

**Table 2:** Exposure to clarithromycin in the first trimester.

Type of major malformation	Number of offspring diagnosed with a major malformation		Odds ratio (CI95%)	
	Exposed N = 253	Unexposed N = 705 584	Unadjusted	adjusted
Congenital malformations of the nervous system	0 (0.0)	886 (0.1)	–	–
Neural Tube Defects	0 (0.0)	292 (0.0)		
Congenital malformations of the eye	0 (0.0)	950 (0.1)	–	–
Congenital malformations of the ear, face and neck	0 (0.0)	439 (0.1)	–	–
Congenital malformations of the heart	2 (0.8)	5286 (0.8)	1.06 (0.26–4.25)	1.04 (0.26–4.17)
Oro-facial clefts	0 (0.0)	1295 (0.2)	–	–
Congenital malformations of the digestive system	0 (0.0)	1277 (0.2)	–	–
Congenital malformations of the internal urinary system	0 (0.0)	1819 (0.3)	–	–
Congenital malformations of the external genital organs	2 (0.8)	2077 (0.3)	2.70 (0.67–10.86)	2.86 (0.71–11.51)
Congenital malformations of the limbs	5 (2.0)	7509 (1.1)	1.88 (0.77–4.55)	1.97 (0.81–4.78)
Congenital malformations of the musculoskeletal system	0 (0.0)	1179 (0.2)	–	–
Chromosomal abnormalities	0 (0.0)	983 (0.1)	–	–
Teratogenic syndromes with malformations	0 (0.0)	63 (0.0)	–	–
Genetic syndromes and microdeletions	0 (0.0)	529 (0.1)	–	–
Other malformations	0 (0.0)	1131 (0.2)	–	–
Congenital malformations of the respiratory system	0 (0.0)	638 (0.1)	–	–
Abdominal wall defects	0 (0.0)	178 (0.0)	–	–
All major congenital malformations	9 (3.6)	24 808 (3.5)	1.01 (0.52–1.97)	1.03 (0.53–2.00)

CI95%, 95% confidence intervals.

### Other Analyses

**Figure 1:**
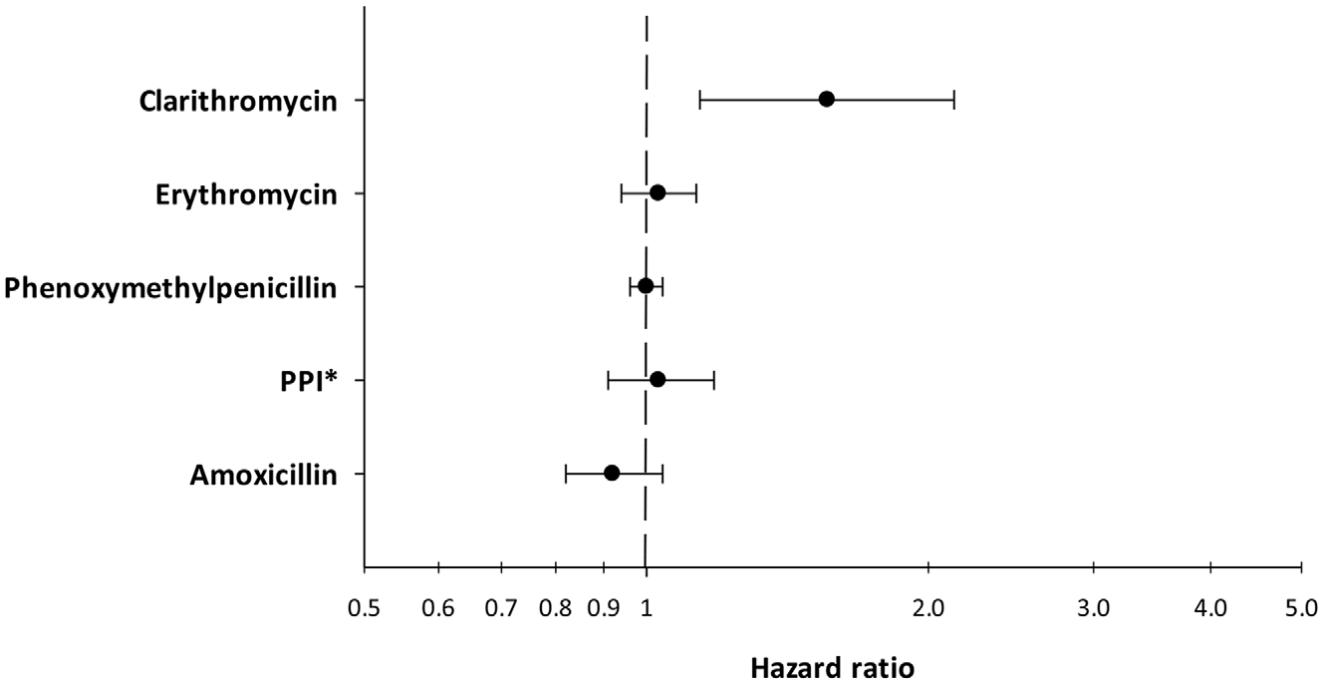
The hazard ratio of having a miscarriage when redeeming a prescription of a drug in the first trimester of pregnancy. All hazards are adjusted for age, parity, educational level, and income. *Proton pump inhibitors.

**Figure 2:**
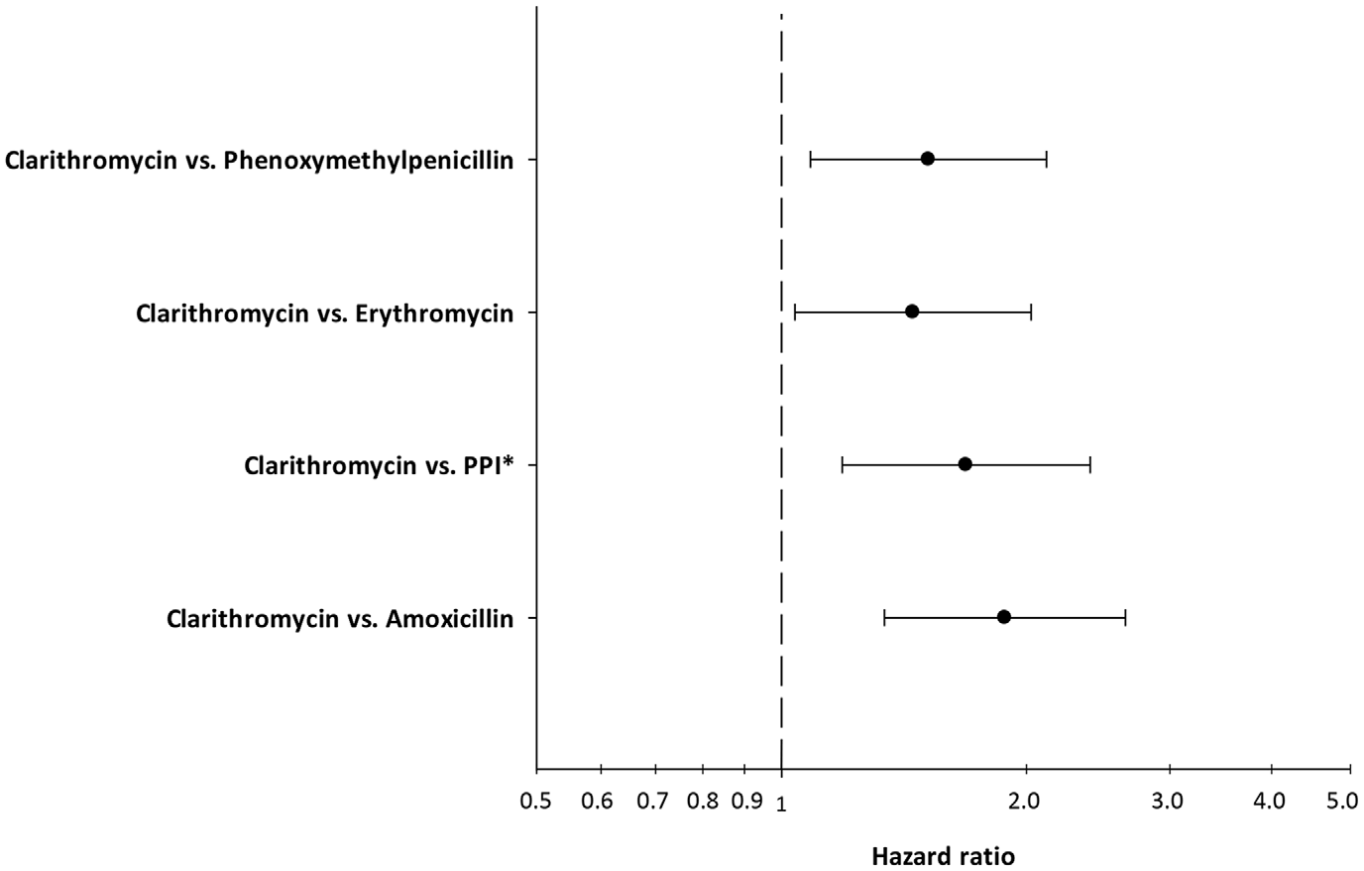
The hazard ratio of having a miscarriage when redeeming a prescription of clarithromycin in the first trimester of pregnancy directly compared to the hazard of women redeeming prescriptions of different drugs used in similar conditions. All hazards are adjusted for age, parity, educational level, and income. *Proton pump inhibitors.

To investigate if the increased hazard of having a miscarriage when exposed to clarithromycin could be confounded by indication, we tested for the hazard of some drugs typically used for respiratory infections in Denmark. We found no increased hazard of having a miscarriage after redeeming a prescription, in the first trimester, of erythromycin (HR = 1.00 (CI95% 0.92–1.10)) or phenoxymethylpenicillin (HR = 1.02 (CI95% 0.98–1.07)). Furthermore, we investigated if use of PPI or amoxicillin, both used as first line treatment in combination with clarithromycin in helicobacter pylori infection, were associated with miscarriages. We found no increased HR for PPI or amoxicillin (table 3, figure 1). Furthermore we estimated the hazard of miscarriage in women exposed to clarithromycin compared directly to women exposed to the drugs used in same conditions as clarithromycin. We found that women exposed to clarithromycin had an adjusted hazard of 1.51 (CI95% 1.09–2.12) of having a miscarriage compared directly to the hazard of miscarriage in women exposed to phenoxymethylpenicillin, and 1.45 (CI95% 1.04–2.03) compared directly to the hazard of miscarriage in women exposed to erythromycin (figure 2). Women exposed clarithromycin had an adjusted hazard of 1.69 (CI95% 1.19–2.39) of having a miscarriage compared directly to women exposed to PPIs and an adjusted hazard of 1.88 (CI95% 1.34–2.65) compared directly to women exposed to amoxicillin (figure 2).

**Table 3:** Sensitivity analyses. Adjusted hazard ratios of having a miscarriage when redeeming a prescription in the first trimester compared to unexposed.

	Number of exposed	Number of unexposed	Hazard ratio unadjusted	Hazard ratio adjusted
Erythromycin	6492	925 012	1.04 (0.95–1.14)	1.03 (0.94–1.13)
Phenoxymethylpenicillin	33 469	898 035	1.01 (0.97–1.06)	1.00 (0.96–1.04)
Proton pump inhibitors	3577	927 927	1.09 (0.96–1.24)	1.03 (0.91–1.18)
Amoxicillin	4584	926 920	0.96 (0.86–1.08)	0.92 (0.82–1.04)

There was no increased hazard of having a miscarriage when being exposed to clarithromycin in the 12 weeks before conception (HR = 0.90 (CI95% 0.77–1.06)).

## Discussion

We found an increased hazard of having a miscarriage when redeeming a prescription of clarithromycin in the first trimester of pregnancy. We did not find an association between exposure to clarithromycin and major congenital malformations.

The findings of this study strengthen other available data. In an animal study an increased number of fetal losses among rabbits given an intravenous dose 17 times lower than the maximum proposed human dose. [1] In monkeys, clarithromycin plasma levels three times the human serum level, resulted in embryonic loss. [1] An observational study showed that women treated with clarithromycin in the first trimester had a doubling of the number of miscarriages compared to a control group. Although the finding was statistical significant compared to the control group the authors noted that the number of miscarriages in the exposed group was within the expected range and they conclude that clarithromycin can be taken safely in pregnancy. [2]

To assess the robustness of our findings we analysed the hazard of having a miscarriage when redeeming a prescription of phenoxymethylpenicillin and erythromycin. These are two antibiotics commonly used in Denmark to treat respiratory infections. We did not find increased hazard ratios of miscarriages in these groups indicating that infections requiring these treatments are not associated with miscarriages nor do the drugs induce miscarriages themselves. It has previously been shown that the transplacental transfer of clarithromycin is two times that of erythromycin. [14], [15] This could potentially explain why we find an increased hazard for clarithromycin but not for erythromycin. Furthermore, we analyzed the hazard of having a miscarriage when redeeming a prescription of PPIs or amoxicillin. Both drugs are used in the treatment of helicobacter pylori infections in combination with clarithromycin. We did not find an increased hazard of miscarriage when redeeming prescriptions on these drugs, which indicates that PPIs and amoxicillin and infections treated with these drugs are not, associated with miscarriages. The hazards of phenoxymethylpenicillin, erythromycin, PPIs and amoxicillin suggest that the primary result regarding clarithromycin and miscarriages are not a product of confounding by indication.

To address the importance of other characteristics of women using clarithromycin, we compared the result with dispensing of clarithromycin much earlier than the pregnancy period but found no increased prevalence with dispensing in the 12 weeks before pregnancy. This indicates that the increased hazard of having a miscarriage is not explained by unaccounted characteristics of exposed women.

We did not find an association between first trimester exposure to clarithromycin and major congenital malformations. All though this is a small study with limited power, the result is supported by three other studies. [2], [16], [17] A higher rate of heart defects has been observed among rat offspring when the mother was given clarithromycin during pregnancy compared to unexposed controls. [1] Likewise, studies on mice demonstrated a higher rate of offspring with cleft palate when given clarithromycin compared to controls. [1] None of the clarithromycin exposed in our study were given a diagnosis of oro-facial cleft and we did not find an increased odds ratio of being diagnosed with a heart defect when exposed to clarithromycin in the first trimester compared to unexposed.

The main limitation of the present study is the lack of information concerning treatment indication and we can therefore not completely rule out that our results are confounded by indications. Furthermore, we do not have information on the prescribed dosage and therefore the analyses are restricted to treatment or no treatment. Even though the women went to the pharmacy, redeemed the prescription and paid for the drug we do not know whether or not they were exposed. If they were not exposed, our results would be biased towards underestimating the effect. Furthermore there is a risk of bias due to unaccounted confounders; even though we analysed the risk miscarriage of women exposed to clarithromycin before pregnancy we cannot completely rule out that women exposed to clarithromycin might differ from unexposed women in aspects causally related to the outcome. This could include obesity, alcohol consumption, smoking and antiphospholipid syndrome.

Another limitation is the low number of women exposed to clarithromycin in the study period. Analyses of risk of different subgroups of major congenital malformations should therefore be interpreted with caution.

We only have information on the date the miscarriage was recognized by the women and unfortunately not the exact time when the fetus perished in the uterus. If the exposure to clarithromycin was before the actual miscarriage, but after the fetus perished, the analyses could be biased.

We used registers to define the outcomes and they may be subject to misclassification. If women experience a miscarriage without contacting a hospital the number of registered miscarriages would be underestimated. This underreporting has been estimated to 30% [18] and is probably due to miscarriages early in pregnancy. We find no indications of a difference in the reporting of miscarriages between the two groups. If the increased hazard of miscarriages in women treated for an infection during the first trimester should be due to better reporting we would expect to find the same increase in the women treated with erythromycin, penicillin and amoxicillin, which we did not (table 3). The quality of the malformation diagnoses has been validated and found to have a predictive value of 88% for having a congenital malformation, with a completeness of 90% and misclassification, if any, was most probably random. [19].

This study covers nationwide data and includes all registered birth, provoked abortions and miscarriages in the study period. We thereby minimize selection bias, and ensure high completeness of data independent of social-, economic-, and educational level, age and race.

The registers have previously been validated and found to be accurately recorded. More than 99.5% of all birth in Denmark have been recorded in the Danish Fertility Database [5], [6] and more than 99% of all discharge diagnoses have been registered in the National Hospital Register. [20].

Exposure is based on information from the National Prescription Register which holds records on all redeemed prescriptions which means that the drugs have been collected at the pharmacy and paid for. Completeness has been estimated to 98%. [12].

This study demonstrates that treatment with clarithromycin in the first trimester of pregnancy was associated with a 50% increase (hazard ratio) of miscarriage compared to no treatment and a similarly increased risk compared to penicillin and erythromycin. We did not find an association between clarithromycin and major congenital malformations. The results are similar to other studies addressing the same issue both in human and animals. However, further research is required to explore the possible effect of treatment indication on the associations found.
